# Crystal structure and Hirshfeld surface analysis of (2*E*,2′*E*)-1,1′-[seleno­bis­(4,1-phenyl­ene)]bis­[3-(4-chloro­phen­yl)prop-2-en-1-one]

**DOI:** 10.1107/S2056989019014038

**Published:** 2019-10-22

**Authors:** Hazem Bouraoui, Youcef Mechehoud, Souheyla Chetioui, Rachid Touzani, Meriem Medjani, Ahmed Benmilat, Ali Boudjada

**Affiliations:** a Laboratoire de Cristallographie, Département de Physique, Université des Frères Mentouri-Constantine, 25000 Constantine, Algeria; b Université de Ouargla, Faculté des Mathématiques et Sciences de la Matiére, Route de Ghardaia, Ouargla 30000, Algeria; c Laboratoire VAREN, Département de Chimie, Faculté des Sciences Exactes, Université Mentouri-Constantine, 25000 Constantine, Algeria; d Unité de Recherche de Chimie de l’Environnement et Moléculaire Structurale (CHEMS), Faculté des Sciences Exactes, Département de Chimie, Université des Frères Mentouri Constantine, Constantine 25000, Algeria; e Faculté de Technologie, Université Mohamed Boudiaf, M’sila, Algeria; f Laboratoire de Chimie Appliquée et Environnement, LCAE-URAC18, COSTE, Faculté des Sciences, Université Mohamed Premier, BP524, 60000, Oujda, Morocco; g Faculté Pluridisciplinaire Nador BP 300, Selouane 62702, Nador, Morocco

**Keywords:** crystal structure, organoselenium, selenium, Hirshfeld surface analysis

## Abstract

In the title com­pound, C_30_H_20_Cl_2_O_2_Se, the C—Se—C angle is 99.0 (2)°, with the dihedral angle between the planes of the attached benzene rings being 79.1 (3)°. The average endocyclic angles (Se—C—C) facing the Se atom are 122.1 (5) and 122.2 (5)°. The Se atom is essentially coplanar with the attached benzene rings, deviating by 0.075 (1) and 0.091 (1) Å. In the two phenyl­ene(4-chloro­phen­yl)prop-2-en-1-one units, the benzene rings are inclined to each other by 44.6 (3) and 7.8 (3)°. In the crystal, the mol­ecules stack up the *a* axis, forming layers parallel to the *ac* plane. There are no significant classical inter­molecular inter­actions present. Hirshfeld surface analysis, two-dimensional fingerprint plots and the mol­ecular electrostatic potential surface were used to analyse the crystal packing. The Hirshfeld surface analysis suggests that the most significant contributions to the crystal packing are by C⋯H/H⋯C contacts (17.7%).

## Abstract 2

In the title organoselenium com­pound, the C—Se—C angle is 99.0 (2)°, with the dihedral angle between the planes of the attached benzene rings being 79.1 (3)°.

## Chemical context   

During the last few years, organoselenium chemistry (Procter, 2001 ▸) has been the subject of constant scientific inter­est and organoselenium com­pounds have been used intensively as important reagents and inter­mediates in organic synthesis (Zade *et al.*, 2005 ▸). Recently, various organoselenium com­pounds have attracted growing attention in medicine. Seleno­proteins are very important for neuronal survival and function. It has been found that seleno­protein P may influence Alzheimer pathology (Bellinger *et al.*, 2008 ▸). Furthermore, the potential of seleno­proteins to protect against oxidative stress led to the expectation that selenium would be protective against type 2 diabetes, and indeed in the 1990s, selenium was shown to have anti­diabetic and insulin mimetic effects (Steinbrenner *et al.*, 2011 ▸). However, more recently, findings from observational epidemiological studies and randomized clinical trials have raised concern that high selenium exposure may lead to type 2 diabetes or insulin resistance at least in well-nourished populations (Stranges *et al.*, 2010 ▸). In addition, mol­ecules involving selenium are still efficient and encouraged in medicinal chemistry (Zhao *et al.*, 2012 ▸). Moreover, organoselenium com­pounds are of considerable inter­est in academia, as anti­cancer (Zhu & Jiang, 2008 ▸), anti-oxidant (Anderson *et al.*, 1996 ▸), anti-inflammatory and anti­allergic agents (Abdel-Hafez, 2008 ▸), and in industry because of their involvement as key inter­mediates in the synthesis of pharmaceuticals (Woods *et al.*, 1993 ▸), fine chemicals and polymers (Hellberg *et al.*, 1997 ▸). Moreover, chalcone derivatives are notable for their excellent blue-light transmittance and good crystallizability; they also show considerable promise as organic nonlinear optical materials (Uchida *et al.*, 1998 ▸). In continuation of our work on chalcone organoselenium derivatives, we report herein on the crystal structure of (2*E*,2′*E*)-1,1′-[seleno­bis­(4,1-phenyl­ene)]bis­[3-(4-chloro­phen­yl)prop-2-en-1-one].
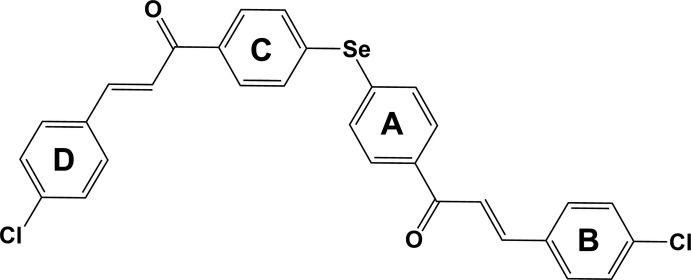


## Structural commentary   

**Figure 1:**
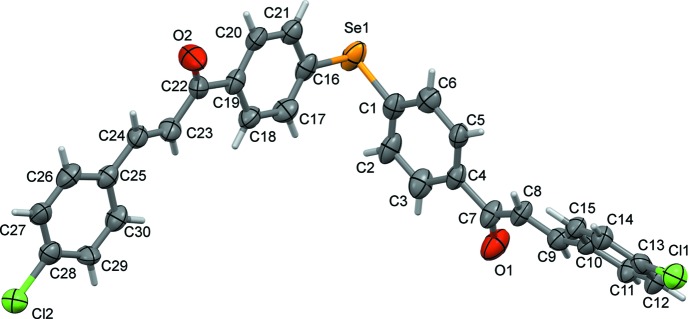
The mol­ecular structure of the title com­pound, with the atom labelling and displacement ellipsoids drawn at the 50% probability level.

The mol­ecular structure of the title com­pound is shown in Fig. 1 ▸. The C1—Se1—C16 angle is 99.0 (2)°, which is close to the value observed in three very similar com­pounds, *viz*. 99.47 (10)° in bis­(4-nitro­phen­yl) selenide, where the Se atom lies on a twofold rotation axis (Zuo, 2013 ▸), 99.59 (14)° in bis­(4-acetyl­phen­yl) selenide (Bouraoui *et al.*, 2011 ▸) and 100.03 (15)° in bis­(2-chloro­ethan-1-one-phen­yl) selenide (Bouraoui *et al.*, 2015 ▸).

In the title com­pound, inner benzene rings *A* (atoms C1–C6) and *C* (C16–C21) (see Scheme) are inclined to each other by 79.1 (3)°. This is similar to the same angle observed for the acetyl­phenyl derivative, *viz*. 87.08 (15)°, but considerably different to that observed for the 4-nitro­phenyl derivative, *viz*. 63.76 (10)°.

In each phenyl­ene-(4-chloro­phen­yl)prop-2-en-1-one unit, the C=C has an *E* configuration. The C=C bond lengths C8=C9 and C23=C24 are 1.317 (8) and 1.325 (8) Å, respectively, which confirms their double-bond character. Benzene rings *A* and *B* (C10–C15) of one unit are inclined to one another by 44.6 (3)°, while rings *C* and *D* (C25–C30) of the other unit are almost coplanar, with a dihedral angle of 7.8 (3)°. The outer benzene rings, *B* and *D*, are almost normal to one another, with a dihedral angle of 84.4 (3)°.

## Supra­molecular features   

**Figure 2:**
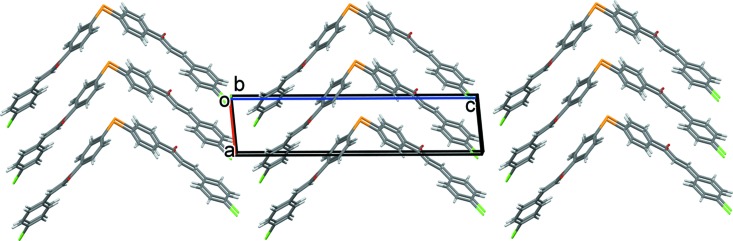
A view along the *b* axis of the crystal packing of the title com­pound, showing the layer-like structure.

**Figure 3:**
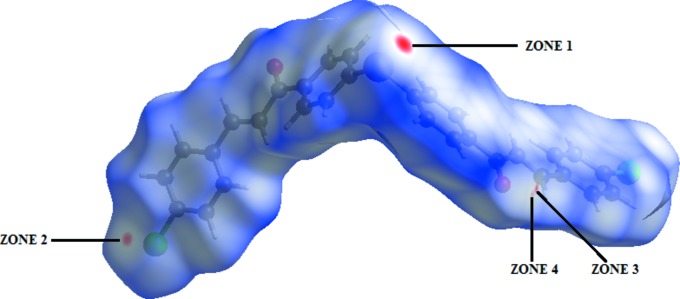
A view of the Hirshfeld surface mapped over *d*_norm_ in the colour range −0.0711 to 1.3645 a.u.

In the crystal, mol­ecules stack up the *a* axis, forming layers parallel to the *ac* plane (Fig. 2 ▸). There are no significant classical inter­molecular inter­actions present (*PLATON*; Spek, 2009 ▸). The shortest atom–atom contacts in the crystal (Figs. 3 ▸ and 4 ▸) are given in Table 1 ▸ and are discussed in §4 (*Hirshfeld surface analysis*).

## Hirshfeld surface analysis   

Insight into the inter­molecular inter­actions in the crystal were obtained from an analysis of the Hirshfeld surface (Spackman & Jayatilaka, 2009 ▸) and the two-dimensional fingerprint plots (McKinnon *et al.*, 2007 ▸). The program *CrystalExplorer* (Turner *et al.*, 2017 ▸) was used to generate both the Hirshfeld surfaces, mapped over *d*_norm_, and the electrostatic potential for the title com­pound. The function *d*_norm_ is a ratio enclosing the distances of any surface point to the nearest inter­ior (*d*_i_) and exterior (*d*_e_) atom and the van der Waals (vdW) radii of the atoms. The function *d*_norm_ will be equal to zero when inter­molecular distances are close to the van der Waals contacts. They are indicated by a white colour on the Hirshfeld surface, while contacts longer than the sum of the vdW radii with positive *d*_norm_ values are coloured blue.

**Figure 4:**
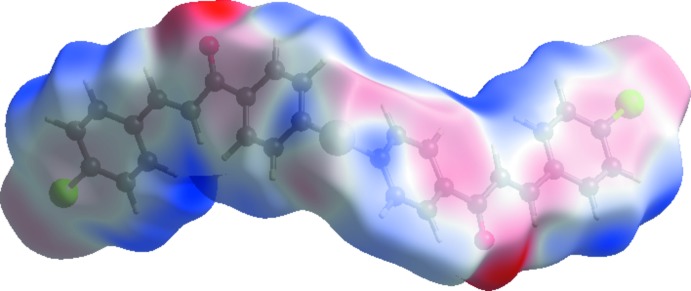
A view of the Hirshfeld surface plotted over the calculated electrostatic potential energy in the range −0.0489 to 0.0448 a.u.

The analysis of the Hirshfeld surface (HS) mapped over *d*_norm_ is shown in Fig. 4 ▸. The H⋯O contacts between the corresponding donor and acceptor atoms are visualized as bright-red spots on the side (zone 4) of the Hirshfeld surface (Fig. 4 ▸). Three other red spots exist, corresponding to the C⋯Se, Cl⋯Cl and C⋯O contacts, *viz*. zones 1, 2 and 3, respectively (Fig. 4 ▸). These contacts are considered to be the strongest when com­paring them to the sum of the vdW radii [Table 1 ▸; calculated using *Mercury* (Macrae *et al.*, 2008 ▸)].

**Figure 5:**
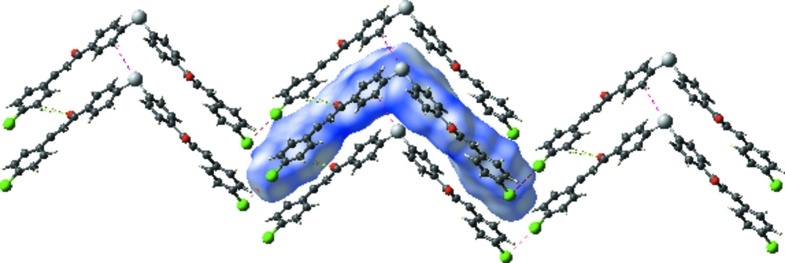
Hirshfeld surface mapped over *d*_norm_ to visualize some of the short inter­molecular contacts in the crystal (see Table 1 ▸).

A view of the mol­ecular electrostatic potential using the 6-31G(d) basis set with the density functional theory (DFT) method for the title com­pound is shown in Fig. 5 ▸. The H⋯O donors and acceptors are shown as blue and red areas around the atoms related with positive (hydrogen-bond donors) and negative (hydrogen-bond acceptors) electrostatic potentials, respectively.

**Figure 6:**
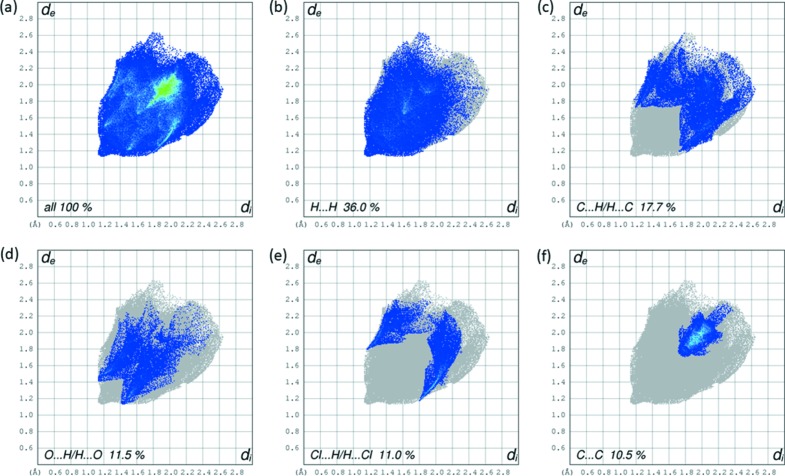
(*a*) The full two-dimensional fingerprint plot for the title com­pound and those delineated into (*b*) H⋯H, (*c*) C⋯H/H⋯C, (*d*) O⋯H/H⋯O, (*e*) Cl⋯H/H⋯Cl and (*f*) C⋯C contacts.

The full two-dimensional fingerprint plot for the title com­pound is given in Fig. 6 ▸(*a*). Those for the most significant contacts contributing to the HS are given in Fig. 6 ▸(*b*) for H⋯H, Fig. 6 ▸(*c*) for C⋯H/H⋯C, Fig. 6 ▸(*d*) for O⋯H/H⋯O, Fig. 6 ▸(*e*) for Cl⋯H/H⋯Cl and Fig. 6 ▸(*f*) for C⋯C. A full list of the relative percentage contributions of the close contacts to the HS of the title com­pound are given in Table 2 ▸.

A contribution of 36.0% was found for the H⋯H contacts (Fig. 6 ▸ *b*), representing the largest contribution, and is displayed on the fingerprint plots by a pair of very short spikes at *d*_e_ + *d*_i_ = 2.3 Å; the vdW radius for this inter­action is 2.18 Å, which means it is a weak inter­action.

The C⋯H/H⋯C (17.7%, Fig. 6 ▸ *c*) and Cl⋯H/H⋯Cl (Fig. 6 ▸ *e*) contacts are seen as pairs of spikes at *d*_e_ + *d*_i_ = 2.9 and 2.9 Å, respectively.

The plot of O⋯H/H⋯O contacts between H atoms located inside the Hirshfeld surface and oxygen from outside and *vice versa* is shown in Fig. 6 ▸(*d*). These contacts account for 11.5% and are characterized by two symmetrical peaks with *d*_e_ + *d*_i_ = 2.5 Å; this reveals the presence of strong O⋯H contacts.

The C⋯C contacts (Fig. 6 ▸ *f*) give a contribution of 10.5%, while the C⋯Cl, C⋯Se, Se⋯H/H⋯Se and Cl⋯Cl contacts in the structure give weak contributions of 4.3, 3.5, 2.8 and 2.4%, respectively, to the Hirshfeld surface.

## Database survey   

A search of the Cambridge Structural Database (CSD, Version 5.40, last update May 2019; Groom *et al.*, 2016 ▸) for 4,4′-substituted bis­(phen­yl) selenides yielded six relevant hits. These are bis­(2-chloro­ethan-1-one-phen­yl) selenide (CSD refcode HUYRUC; Bouraoui *et al.*, 2015 ▸), bis­(4-nitro­phen­yl) selenide (IDIOG; Zuo, 2013 ▸), bis­(4-meth­oxy­phen­yl) selenide (LAFNAK; Verma *et al.*, 2016 ▸), bis­(4-acetyl­phen­yl) selenide (UPAGAU; Bouraoui *et al.*, 2011 ▸), bis­(phen­yl) selenide itself (YEWYUX; Bhandary *et al.*, 2018 ▸) and bis­(*p*-tol­yl) selenide (TOLYSE; Blackmore & Abrahams, 1955 ▸). In IDIOG, the Se atom lies on a twofold rotation axis, and only YEWYUX and TOLYSE crystallize in chiral space groups, *i.e. P*2_1_ and *P*2_1_2_1_2_1_, respectively.

In the title com­pound (Fig. 1 ▸), the C—Se—C angle is 99.0 (2)°, similar to the value observed in five of the com­pounds mentioned above, *viz.* 100.03 (15), 99.47 (10), 102.25 (19), 99.59 (14) and 98.31 (16)° for HUYRUQ, IDITOG, LAFNAK, UPAGAU and YEWYUX, respectively. In the sixth com­pound, TOLYSE, the dihedral angle is 105.65 (19)°. The two inner benzene rings, *A* and *C*, in the title com­pound are inclined to each other by 79.1 (3)°. This value is quite different to that observed in the five com­pounds mentioned above, *i.e.* 69.92 (17), 63.76 (10), 69.6 (2), 87.08 (15), 68.46 (18) and *ca* 56.99° for HUYRUQ, IDITOG, LAFNAK, UPAGAU, YEWYUX and TOLYSE, respectively.

## Synthesis and crystallization   

The title com­pound was prepared according to a method proposed by Mechehoud *et al.* (2010 ▸). 2-Chloro-1-(4-chloro­phen­yl)ethan-1-one (ClC_8_H_6_COCl; 36.5 mmol) and anhydrous aluminium chloride (5 g, 37.5 mmol, 3 equiv.) were taken up in dry methyl­ene chloride (100 ml). The reaction mixture was cooled to 273–278 K, protected from atmospheric moisture and stirred continuously for 15 min. A solution of diphenyl selenide (3 g, 1.87 mmol) in CH_2_Cl_2_ was added dropwise over a period of 5 min. The reaction mixture was allowed to reach room temperature gradually and then stirred at this temperature overnight. The solution was then washed with ice water–HCl (80 ml) and extracted with CH_2_Cl_2_. The organic layer was separated and dried (Na_2_SO_4_). Removal of the solvent under reduced pressure afforded the crude product, which was recrystallized from petroleum ether to yield 4.2 g of the title com­pound. Yellow single crystals suitable for X-ray diffraction analysis were obtained by recrystallization from CH_2_Cl_2_.

## Refinement   

Crystal data, data collection and structure refinement details are summarized in Table 3 ▸. The H atoms could all be located in a difference Fourier map. During refinement, they were included in calculated positions and refined as riding on the parent C atom, with C—H = 0.93 Å and *U*_iso_(H) = 1.2*U*_eq_(C).

## Supplementary Material

Crystal structure: contains datablock(s) Global, I. DOI: 10.1107/S2056989019014038/su5517sup1.cif

CCDC references: 1959404, 1959404

Additional supporting information:  crystallographic information; 3D view; checkCIF report

## Figures and Tables

**Table 1:** Short contacts (Å) in the crystal of the title com­pound

Atom 1	Atom 2	Length (Å)	vdW length (Å)
H3	H10^i^	2.498	0.098
O2	H19^ii^	2.632	−0.088
H12	O2^iii^	2.759	0.039
O1	H3^iii^	2.770	0.050
O2	H20^ii^	2.818	0.098
H2	C6^iii^	2.922	0.022
C3	H4^ii^	2.943	0.043
H3	C15^i^	2.964	0.064
O2	C29^ii^	3.217	−0.003
O2	C30^ii^	3.314	0.094
C5	C8^i^	3.461	0.061
Se1	C17^i^	3.475	−0.125
C20	C23^i^	3.480	0.080
Cl2	Cl1^iv^	3.549	0.049

Symmetry codes: (i) *x* − 1, *y*, *z*; (ii) *x* − 1, *y* + 1, *z*; (iii) *x*, *y* − 1, *z*; (iv) *x* − 1, *y* − 1, *z* + 1.

**Table 2:** Relative percentage contributions of the close contacts to the Hirshfeld surface of the title com­pound

Contact	Percentage contribution
H⋯H	36.0
C⋯H/H⋯C	17.7
O⋯H/H⋯O	11.5
Cl⋯H/H⋯Cl	11.0
C⋯C	10.5
C⋯Cl	4.3
C⋯Se	3.5
Se⋯H/H⋯Se	2.8
Cl⋯Cl	2.4
C⋯O	0.3

**Table 3:** Experimental details

Crystal data	
Chemical formula	C_30_H_20_Cl_2_O_2_Se
*M*_r_	562.32
Crystal system, space group	Triclinic, *P*1
Temperature (K)	293
*a*, *b*, *c* (Å)	4.9468 (3), 5.8712 (6), 21.3530 (18)
α, β, γ (°)	85.019 (8), 84.094 (6), 86.465 (7)
*V* (Å^3^)	613.68 (9)
*Z*	1
Radiation type	Mo *K*α
μ (mm^−1^)	1.77
Crystal size (mm)	0.03 × 0.02 × 0.01

Data collection	
Diffractometer	Agilent Technologies Xcalibur Eos
No. of measured, independent and observed [*I* > 2σ(*I*)] reflections	5341, 3672, 2465
*R*_int_	0.030
(sin θ/λ)_max_ (Å^−1^)	0.661

Refinement	
*R*[*F* ^2^ > 2σ(*F* ^2^)], *wR*(*F* ^2^), *S*	0.038, 0.074, 0.81
No. of reflections	3672
No. of parameters	317
No. of restraints	3
H-atom treatment	H-atom parameters constrained
Δρ_max_, Δρ_min_ (e Å^−3^)	0.41, −0.32
Absolute structure	Refined as an inversion twin
Absolute structure parameter	0.002 (11)

Computer programs: *CrysAlis PRO* (Agilent, 2013 ▸), *SHELXS97* (Sheldrick, 2008 ▸), *Mercury* (Macrae *et al.*, 2008 ▸), *SHELXL2018* (Sheldrick, 2015 ▸), *PLATON* (Spek, 2009 ▸) and *publCIF* (Westrip, 2010 ▸).

## supplementary crystallographic information

### Crystal data

**Table:**

C_30_H_20_Cl_2_O_2_Se	*Z* = 1
*M**_r_* = 562.32	*F*(000) = 284
Triclinic, *P*1	*D*_x_ = 1.522 Mg m^−^^3^
*a* = 4.9468 (3) Å	Mo *K*α radiation, λ = 0.71073 Å
*b* = 5.8712 (6) Å	Cell parameters from 1538 reflections
*c* = 21.3530 (18) Å	θ = 3.9–28.9°
α = 85.019 (8)°	µ = 1.77 mm^−^^1^
β = 84.094 (6)°	*T* = 293 K
γ = 86.465 (7)°	Prism, yellow
*V* = 613.68 (9) Å^3^	0.03 × 0.02 × 0.01 mm

### Data collection

**Table:**

Agilent Technologies Xcalibur Eos diffractometer	*R*_int_ = 0.030
Graphite monochromator	θ_max_ = 28.0°, θ_min_ = 2.9°
ω scans	*h* = −6→6
5341 measured reflections	*k* = −7→5
3672 independent reflections	*l* = −28→28
2465 reflections with *I* > 2σ(*I*)	

### Refinement

**Table:**

Refinement on *F*^2^	Secondary atom site location: difference Fourier map
Least-squares matrix: full	Hydrogen site location: inferred from neighbouring sites
*R*[*F*^2^ > 2σ(*F*^2^)] = 0.038	H-atom parameters constrained
*wR*(*F*^2^) = 0.074	*w* = 1/[σ^2^(*F*_o_^2^) + (0.0181*P*)^2^] where *P* = (*F*_o_^2^ + 2*F*_c_^2^)/3
*S* = 0.81	(Δ/σ)_max_ < 0.001
3672 reflections	Δρ_max_ = 0.41 e Å^−^^3^
317 parameters	Δρ_min_ = −0.32 e Å^−^^3^
3 restraints	Absolute structure: Refined as an inversion twin
Primary atom site location: structure-invariant direct methods	Absolute structure parameter: 0.002 (11)

### Special details

**Table:**

Geometry. All esds (except the esd in the dihedral angle between two l.s. planes) are estimated using the full covariance matrix. The cell esds are taken into account individually in the estimation of esds in distances, angles and torsion angles; correlations between esds in cell parameters are only used when they are defined by crystal symmetry. An approximate (isotropic) treatment of cell esds is used for estimating esds involving l.s. planes.
Refinement. Refined as a 2-component inversion twin.

### Fractional atomic coordinates and isotropic or equivalent isotropic displacement parameters (Å^2^)

**Table:**

	*x*	*y*	*z*	*U*_iso_*/*U*_eq_	
Se1	0.41094 (7)	0.32993 (9)	0.51703 (4)	0.0688 (2)	
Cl1	2.5172 (3)	0.7697 (3)	0.08312 (8)	0.0590 (4)	
Cl2	2.0618 (3)	−0.0037 (3)	0.97993 (8)	0.0649 (5)	
O1	1.4087 (9)	−0.1409 (9)	0.3126 (3)	0.0780 (15)	
O2	0.9496 (10)	0.8409 (8)	0.7498 (2)	0.0823 (16)	
C1	0.7186 (10)	0.2486 (11)	0.4597 (3)	0.0479 (15)	
C2	0.8352 (11)	0.0295 (12)	0.4605 (3)	0.0615 (18)	
H1	0.768523	−0.081384	0.490939	0.074*	
C3	1.0483 (12)	−0.0293 (12)	0.4171 (3)	0.0593 (17)	
H2	1.122475	−0.178850	0.418221	0.071*	
C4	1.1520 (10)	0.1346 (11)	0.3718 (3)	0.0444 (14)	
C5	1.0451 (11)	0.3551 (11)	0.3723 (3)	0.0503 (16)	
H3	1.120388	0.468932	0.343979	0.060*	
C6	0.8227 (12)	0.4092 (12)	0.4153 (3)	0.0553 (18)	
H4	0.744240	0.557370	0.413526	0.066*	
C7	1.3680 (11)	0.0604 (11)	0.3220 (3)	0.0507 (15)	
C8	1.5292 (10)	0.2372 (11)	0.2840 (3)	0.0461 (15)	
H5	1.522998	0.383771	0.297588	0.055*	
C9	1.6809 (10)	0.1939 (11)	0.2315 (3)	0.0457 (15)	
H6	1.666213	0.051207	0.216721	0.055*	
C10	1.8698 (9)	0.3462 (10)	0.1941 (3)	0.0439 (14)	
C11	2.0024 (10)	0.2799 (11)	0.1368 (3)	0.0499 (15)	
H7	1.958306	0.143756	0.122056	0.060*	
C12	2.1960 (11)	0.4092 (11)	0.1015 (3)	0.0534 (16)	
H8	2.277520	0.364813	0.062900	0.064*	
C13	2.2653 (10)	0.6084 (11)	0.1255 (3)	0.0464 (15)	
C14	2.1353 (11)	0.6771 (12)	0.1810 (3)	0.0479 (15)	
H9	2.180794	0.813002	0.195644	0.057*	
C15	1.9411 (10)	0.5512 (11)	0.2153 (3)	0.0484 (15)	
H10	1.855730	0.601551	0.252860	0.058*	
C16	0.5992 (10)	0.4225 (11)	0.5837 (3)	0.0498 (16)	
C17	0.7975 (12)	0.2824 (11)	0.6111 (3)	0.0577 (18)	
H11	0.848615	0.141757	0.595285	0.069*	
C18	0.9211 (12)	0.3470 (11)	0.6615 (3)	0.0542 (16)	
H12	1.051962	0.248547	0.679406	0.065*	
C19	0.8519 (11)	0.5582 (11)	0.6859 (3)	0.0457 (14)	
C20	0.6508 (12)	0.6967 (12)	0.6588 (3)	0.0580 (17)	
H13	0.596947	0.836179	0.675061	0.070*	
C21	0.5295 (10)	0.6326 (11)	0.6087 (3)	0.0509 (15)	
H14	0.398311	0.731000	0.590920	0.061*	
C22	0.9847 (12)	0.6393 (11)	0.7381 (3)	0.0540 (16)	
C23	1.1622 (12)	0.4833 (11)	0.7744 (3)	0.0510 (16)	
H15	1.186238	0.331934	0.764354	0.061*	
C24	1.2909 (10)	0.5463 (11)	0.8210 (3)	0.0499 (15)	
H16	1.258745	0.697969	0.830360	0.060*	
C25	1.4776 (10)	0.4073 (10)	0.8597 (3)	0.0453 (14)	
C26	1.5525 (11)	0.4873 (11)	0.9142 (3)	0.0574 (17)	
H17	1.481261	0.629389	0.925872	0.069*	
C27	1.7295 (11)	0.3629 (12)	0.9516 (3)	0.0553 (16)	
H18	1.775600	0.419691	0.988224	0.066*	
C28	1.8357 (10)	0.1557 (11)	0.9342 (3)	0.0505 (16)	
C29	1.7647 (11)	0.0694 (11)	0.8801 (3)	0.0529 (17)	
H19	1.836285	−0.073216	0.868869	0.063*	
C30	1.5893 (10)	0.1942 (10)	0.8431 (3)	0.0522 (16)	
H20	1.544135	0.136366	0.806609	0.063*	

### Atomic displacement parameters (Å^2^)

**Table:**

	*U*^11^	*U*^22^	*U*^33^	*U*^12^	*U*^13^	*U*^23^
Se1	0.0439 (3)	0.1060 (6)	0.0592 (4)	−0.0158 (3)	0.0067 (3)	−0.0279 (4)
Cl1	0.0504 (8)	0.0624 (11)	0.0618 (11)	−0.0106 (7)	0.0025 (7)	0.0047 (9)
Cl2	0.0606 (9)	0.0696 (12)	0.0622 (11)	0.0083 (9)	−0.0051 (8)	−0.0008 (10)
O1	0.101 (4)	0.052 (3)	0.075 (4)	−0.015 (3)	0.031 (3)	−0.013 (3)
O2	0.130 (4)	0.054 (3)	0.068 (3)	0.025 (3)	−0.035 (3)	−0.024 (3)
C1	0.038 (3)	0.067 (5)	0.042 (4)	−0.014 (3)	−0.004 (2)	−0.013 (3)
C2	0.061 (4)	0.075 (5)	0.047 (4)	−0.024 (4)	0.017 (3)	−0.010 (4)
C3	0.066 (4)	0.053 (4)	0.057 (5)	−0.020 (3)	0.011 (3)	−0.004 (4)
C4	0.040 (3)	0.060 (4)	0.036 (3)	−0.012 (3)	−0.003 (2)	−0.014 (3)
C5	0.057 (4)	0.059 (4)	0.033 (4)	−0.009 (3)	0.004 (3)	−0.002 (3)
C6	0.051 (4)	0.061 (5)	0.057 (5)	−0.010 (3)	−0.004 (3)	−0.019 (4)
C7	0.056 (3)	0.054 (4)	0.043 (4)	−0.016 (3)	0.007 (3)	−0.013 (3)
C8	0.046 (3)	0.053 (4)	0.039 (4)	−0.007 (3)	0.006 (3)	−0.016 (3)
C9	0.041 (3)	0.050 (4)	0.046 (4)	0.002 (3)	−0.004 (3)	−0.002 (3)
C10	0.039 (3)	0.048 (4)	0.045 (3)	0.003 (3)	0.000 (2)	−0.010 (3)
C11	0.060 (4)	0.046 (4)	0.045 (4)	−0.002 (3)	0.002 (3)	−0.017 (3)
C12	0.056 (4)	0.063 (5)	0.040 (4)	−0.012 (3)	0.012 (3)	−0.011 (3)
C13	0.037 (3)	0.056 (4)	0.042 (3)	0.007 (3)	−0.003 (2)	0.012 (3)
C14	0.053 (3)	0.050 (4)	0.040 (4)	−0.003 (3)	−0.001 (3)	−0.004 (3)
C15	0.048 (3)	0.059 (4)	0.038 (3)	0.002 (3)	0.002 (3)	−0.013 (3)
C16	0.036 (3)	0.062 (4)	0.050 (4)	−0.019 (3)	0.013 (3)	−0.010 (3)
C17	0.061 (4)	0.047 (4)	0.065 (5)	−0.003 (3)	0.007 (3)	−0.016 (4)
C18	0.064 (4)	0.053 (4)	0.045 (4)	−0.008 (3)	0.006 (3)	−0.012 (3)
C19	0.049 (3)	0.050 (4)	0.036 (3)	−0.003 (3)	0.006 (3)	−0.005 (3)
C20	0.064 (4)	0.059 (4)	0.049 (4)	0.002 (3)	0.009 (3)	−0.014 (3)
C21	0.045 (3)	0.060 (4)	0.047 (4)	0.000 (3)	0.003 (3)	−0.009 (3)
C22	0.066 (4)	0.054 (4)	0.037 (3)	0.016 (3)	0.004 (3)	−0.003 (3)
C23	0.075 (4)	0.038 (4)	0.039 (4)	−0.003 (3)	0.004 (3)	−0.002 (3)
C24	0.056 (3)	0.049 (4)	0.042 (3)	0.002 (3)	0.006 (3)	−0.004 (3)
C25	0.045 (3)	0.046 (4)	0.044 (4)	−0.005 (3)	0.006 (3)	−0.009 (3)
C26	0.059 (4)	0.052 (4)	0.061 (5)	0.000 (3)	0.006 (3)	−0.015 (4)
C27	0.050 (3)	0.069 (5)	0.047 (4)	0.008 (3)	−0.002 (3)	−0.019 (3)
C28	0.040 (3)	0.059 (4)	0.049 (4)	−0.002 (3)	0.006 (3)	0.001 (3)
C29	0.060 (4)	0.042 (4)	0.057 (4)	0.016 (3)	−0.012 (3)	−0.012 (3)
C30	0.056 (3)	0.052 (4)	0.050 (4)	−0.004 (3)	−0.002 (3)	−0.020 (3)

### Geometric parameters (Å, º)

**Table:**

Se1—C1	1.916 (5)	C14—C15	1.362 (8)
Se1—C16	1.913 (6)	C14—H9	0.9300
Cl1—C13	1.741 (6)	C15—H10	0.9300
Cl2—C28	1.737 (6)	C16—C17	1.385 (8)
O1—C7	1.217 (7)	C16—C21	1.396 (8)
O2—C22	1.229 (7)	C17—C18	1.383 (9)
C1—C2	1.376 (8)	C17—H11	0.9300
C1—C6	1.364 (8)	C18—C19	1.396 (8)
C2—C3	1.377 (8)	C18—H12	0.9300
C2—H1	0.9300	C19—C20	1.387 (7)
C3—C4	1.386 (8)	C19—C22	1.477 (8)
C3—H2	0.9300	C20—C21	1.371 (8)
C4—C5	1.368 (8)	C20—H13	0.9300
C4—C7	1.500 (7)	C21—H14	0.9300
C5—C6	1.398 (8)	C22—C23	1.459 (7)
C5—H3	0.9300	C23—C24	1.325 (8)
C6—H4	0.9300	C23—H15	0.9300
C7—C8	1.482 (8)	C24—C25	1.466 (7)
C8—C9	1.317 (8)	C24—H16	0.9300
C8—H5	0.9300	C25—C26	1.383 (8)
C9—C10	1.462 (8)	C25—C30	1.395 (7)
C9—H6	0.9300	C26—C27	1.380 (8)
C10—C11	1.401 (7)	C26—H17	0.9300
C10—C15	1.400 (8)	C27—C28	1.361 (8)
C11—C12	1.380 (7)	C27—H18	0.9300
C11—H7	0.9300	C28—C29	1.387 (8)
C12—C13	1.392 (8)	C29—C30	1.369 (7)
C12—H8	0.9300	C29—H19	0.9300
C13—C14	1.369 (8)	C30—H20	0.9300
			
C1—Se1—C16	99.0 (2)	C17—C16—C21	117.4 (6)
C2—C1—C6	118.2 (5)	C17—C16—Se1	122.2 (5)
C2—C1—Se1	122.1 (5)	C21—C16—Se1	120.3 (5)
C6—C1—Se1	119.7 (5)	C18—C17—C16	121.4 (6)
C1—C2—C3	121.5 (6)	C18—C17—H11	119.3
C1—C2—H1	119.2	C16—C17—H11	119.3
C3—C2—H1	119.2	C17—C18—C19	120.8 (6)
C2—C3—C4	120.0 (6)	C17—C18—H12	119.6
C2—C3—H2	120.0	C19—C18—H12	119.6
C4—C3—H2	120.0	C20—C19—C18	117.6 (6)
C5—C4—C3	119.0 (5)	C20—C19—C22	119.4 (6)
C5—C4—C7	122.4 (5)	C18—C19—C22	123.0 (6)
C3—C4—C7	118.5 (6)	C21—C20—C19	121.5 (6)
C4—C5—C6	120.0 (6)	C21—C20—H13	119.3
C4—C5—H3	120.0	C19—C20—H13	119.3
C6—C5—H3	120.0	C20—C21—C16	121.3 (6)
C1—C6—C5	121.2 (6)	C20—C21—H14	119.4
C1—C6—H4	119.4	C16—C21—H14	119.4
C5—C6—H4	119.4	O2—C22—C19	119.4 (6)
O1—C7—C8	120.5 (6)	O2—C22—C23	120.2 (6)
O1—C7—C4	120.7 (6)	C19—C22—C23	120.4 (6)
C8—C7—C4	118.7 (6)	C24—C23—C22	123.3 (6)
C9—C8—C7	122.3 (6)	C24—C23—H15	118.3
C9—C8—H5	118.9	C22—C23—H15	118.3
C7—C8—H5	118.9	C23—C24—C25	128.2 (6)
C8—C9—C10	127.0 (6)	C23—C24—H16	115.9
C8—C9—H6	116.5	C25—C24—H16	115.9
C10—C9—H6	116.5	C26—C25—C30	117.6 (5)
C11—C10—C15	117.6 (6)	C26—C25—C24	120.2 (6)
C11—C10—C9	119.8 (6)	C30—C25—C24	122.2 (6)
C15—C10—C9	122.5 (6)	C27—C26—C25	122.1 (6)
C12—C11—C10	122.4 (6)	C27—C26—H17	118.9
C12—C11—H7	118.8	C25—C26—H17	118.9
C10—C11—H7	118.8	C28—C27—C26	119.0 (6)
C13—C12—C11	117.8 (6)	C28—C27—H18	120.5
C13—C12—H8	121.1	C26—C27—H18	120.5
C11—C12—H8	121.1	C27—C28—C29	120.6 (5)
C12—C13—C14	120.5 (6)	C27—C28—Cl2	120.1 (5)
C12—C13—Cl1	118.7 (5)	C29—C28—Cl2	119.3 (5)
C14—C13—Cl1	120.8 (6)	C30—C29—C28	120.0 (6)
C15—C14—C13	121.6 (7)	C30—C29—H19	120.0
C15—C14—H9	119.2	C28—C29—H19	120.0
C13—C14—H9	119.2	C29—C30—C25	120.7 (6)
C14—C15—C10	120.1 (6)	C29—C30—H20	119.7
C14—C15—H10	120.0	C25—C30—H20	119.7
C10—C15—H10	120.0		
			
C6—C1—C2—C3	−1.1 (9)	C21—C16—C17—C18	0.4 (9)
Se1—C1—C2—C3	176.9 (5)	Se1—C16—C17—C18	−176.5 (5)
C1—C2—C3—C4	0.7 (10)	C16—C17—C18—C19	−0.9 (10)
C2—C3—C4—C5	2.0 (9)	C17—C18—C19—C20	1.6 (9)
C2—C3—C4—C7	−174.9 (6)	C17—C18—C19—C22	−177.7 (6)
C3—C4—C5—C6	−4.2 (9)	C18—C19—C20—C21	−2.0 (9)
C7—C4—C5—C6	172.5 (6)	C22—C19—C20—C21	177.4 (5)
C2—C1—C6—C5	−1.3 (9)	C19—C20—C21—C16	1.5 (9)
Se1—C1—C6—C5	−179.3 (5)	C17—C16—C21—C20	−0.7 (8)
C4—C5—C6—C1	4.0 (10)	Se1—C16—C21—C20	176.2 (4)
C5—C4—C7—O1	−160.6 (6)	C20—C19—C22—O2	−12.2 (9)
C3—C4—C7—O1	16.1 (9)	C18—C19—C22—O2	167.1 (6)
C5—C4—C7—C8	18.7 (8)	C20—C19—C22—C23	169.6 (6)
C3—C4—C7—C8	−164.5 (5)	C18—C19—C22—C23	−11.1 (9)
O1—C7—C8—C9	13.7 (10)	O2—C22—C23—C24	0.5 (10)
C4—C7—C8—C9	−165.7 (5)	C19—C22—C23—C24	178.8 (5)
C7—C8—C9—C10	−172.9 (5)	C22—C23—C24—C25	−178.6 (5)
C8—C9—C10—C11	−175.5 (6)	C23—C24—C25—C26	−167.4 (6)
C8—C9—C10—C15	9.4 (8)	C23—C24—C25—C30	13.7 (9)
C15—C10—C11—C12	−0.3 (8)	C30—C25—C26—C27	−0.5 (9)
C9—C10—C11—C12	−175.6 (5)	C24—C25—C26—C27	−179.4 (5)
C10—C11—C12—C13	2.1 (8)	C25—C26—C27—C28	0.6 (10)
C11—C12—C13—C14	−2.9 (8)	C26—C27—C28—C29	−0.8 (9)
C11—C12—C13—Cl1	177.9 (4)	C26—C27—C28—Cl2	179.3 (5)
C12—C13—C14—C15	1.9 (9)	C27—C28—C29—C30	0.9 (9)
Cl1—C13—C14—C15	−178.9 (4)	Cl2—C28—C29—C30	−179.2 (5)
C13—C14—C15—C10	0.0 (8)	C28—C29—C30—C25	−0.8 (9)
C11—C10—C15—C14	−0.8 (8)	C26—C25—C30—C29	0.6 (9)
C9—C10—C15—C14	174.4 (5)	C24—C25—C30—C29	179.5 (5)

### Hydrogen-bond geometry (Å, º)

**Table:**

*D*—H···*A*	*D*—H	H···*A*	*D*···*A*	*D*—H···*A*
C29—H19···O2^i^	0.93	2.63	3.218 (8)	122

Symmetry code: (i) *x*+1, *y*−1, *z*.
